# Cafeteria Diet-Induced Metabolic and Cardiovascular Changes in Rats: The Role of *Piper nigrum* Leaf Extract

**DOI:** 10.1155/2021/5585650

**Published:** 2021-06-01

**Authors:** Dorothee L. E. Mballa, Fanta S. A. Yadang, Armelle D. Tchamgoue, Jean R. Mba, Lauve R. Y. Tchokouaha, Emmanuel M. Biang, Alembert T. Tchinda, Désiré P. Djomeni Dzeufiet, Gabriel A. Agbor

**Affiliations:** ^1^Department of Animal Biology, Faculty of Sciences, University of Yaoundé I, Yaoundé, Cameroon; ^2^Centre for Research on Medicinal Plants and Traditional Medicine, Institute of Medical Research and Medicinal Plants Studies, P.O. Box 13033, Yaoundé, Cameroon

## Abstract

**Background:**

Cafeteria diet is known to induce excessive body fat accumulation (obesity) that could cause metabolic and cardiovascular changes and even death. The increase in prevalence over time and the failure in treatment options make obesity a real public health problem. The present study assessed the preventive effect of the hydro-ethanolic extract of the *Piper nigrum* leaf on the development of metabolic and cardiovascular changes in cafeteria diet fed Wistar rats.

**Methods:**

Thirty-six male rats were divided into 5 groups of 6 rats each: a normal control group (Nor.), a negative control group (Neg.), two groups administered different doses of extract in mg/kg (E250 and E500), and a group administered atorvastatin 10 mg/kg (Ator., reference drug). The animals were fed with experimental diets (standard and cafeteria) for a period of 5 weeks. Food and water intake were assessed daily, and the body weight assessed weekly. At the end of the feeding, plasma lipid profile and markers of hepatic and renal function were assessed. Furthermore, the relative weights of the adipose tissue and the organs were assessed. The liver, kidneys, and heart homogenates were assessed for markers of oxidative stress while the aorta was histopathologically examined.

**Results:**

Cafeteria diet-induced weight gain of 30% and increased triglyceride, total cholesterol, and low-density lipoprotein cholesterol level of more than 50%. Equally, an increase in the relative weight of accumulated adipose tissues of more than 90%, oxidative stress, and alteration in the organ structure were visible in cafeteria diet fed rats (Neg). Treatment with *P. nigrum* extract significantly prevented weight gain, dyslipidemia, oxidative stress, and alteration in the architecture of the aorta. The effect of *P. nigrum* extract was comparable to that of the reference drug.

**Conclusion:**

*Piper nigrum* leaf may prevent weight gain and possess cardioprotective activity with a strong antioxidant activity.

## 1. Background

Abnormal accumulation of body fat with health risks defines obesity which is becoming a real public health problem as people are adopting cafeteria diets feeding habits. Obesity is a global epidemic with more than 650 million cases globally between 1975 and 2016 representing approximately 13% of the world's adult population [1]. In Africa, obesity has reached alarming proportions, especially among children where the number of cases increased by almost 50% since the 2000s [1]. Behavioural changes towards high fat diet and decrease in physical exercise contribute to imbalance of energy between excess calories intake and energy spent [2, 3]. Hence, obesity occurs when energy intake surpasses energy expenditure resulting to large energy stores as body fat particularly in the adipose tissues. Accumulation of body fat induces accumulation of adipocytes (hyperplasia) and their size (hypertrophy) bringing about structural and functional changes, activation of inflammatory signalling pathways [4, 5], and discomfort (depression and rejection) [6]. Obesity is a major risk factor for several chronic diseases, including diabetes, cardiovascular diseases, and some types of cancers [7] which not only cause a decline in quality of life but also cause serious complications and premature death [8]. Notwithstanding the multifactorial aetiology of obesity, the prevalence is on the rise with environmental and behavioural factors (including dietary habits) being the major contributors rather than genetic variation [9].

Obesity has been related to induction of oxidative stress with resulting complications like endothelial dysfunction, non-alcoholic fatty liver disease, microvascular complications, and nephropathy [10, 11] and cardiovascular diseases [12]. Obese subjects generally possess low antioxidant defense with enhanced levels of reactive oxygen species (ROS) and reactive nitrogen species (RNS) [13]. More so, in the case of central obesity, the antioxidant mechanism decreases further in an inversely proportional fashion. Obesity related hepatic inflammation is associated with tumour formation in dietary induced obesity in mice [14]. Ulla et al. [13] reported an increase in oxidative stress in experimental animals fed cafeteria diet characterized by an increase in lipid peroxidation and a collapse in antioxidant defense enzymes (catalase, superoxide dismutase, and glutathione peroxidase). Meanwhile, Udomkasemsab et al. [12] reported the implication of cafeteria diet in inducing inflammatory and oxidative stress markers. For this reason, plants that possess antioxidant and anti-inflammatory properties are thought to possess anti-obesity activities as shown in the studies of Ulla et al. [13] and Udomkasemsab et al. [12]. Such medicinal plants contain a wide variety of bioactive components that have beneficial effects on body metabolism and fat oxidation [15].

Currently, many strategies such as increase in physical activities, healthy dietary habits, anti-obesity drugs, and in extreme cases surgery have been put in place for the management of obesity. However, in most cases, these measures do not help because of the complexity of the pathology, cost, and adverse effects of certain pharmacological protocols. Sometimes only 5 to 10% of obese subjects maintain normal weight with a healthy lifestyle [15]. Hence, management of obesity in an efficient way remains a real challenge for the pharmacological industries and the health services. This has challenged many researchers to study medicinal plants and medicinal foods that can limit weight gain in order to prevent obesity. Animal models for obesity were developed by feeding rodents a semi-purified diet for several weeks with a fat content of more than 40% energy based on animal fats and the rodents developed obesity, hyperglycaemia, hypertriglyceridemia, and hyperleptinemia mimicking the pathophysiology of human obesity and metabolic syndrome [9, 16, 17]. Hence, diet-induced obesity models have become one of the most frequently used in studying obesity with high fat and cafeteria diets often employed in rats which have exhibited evidence of the role of over nutrition in energy homeostasis, body weight regulation, and adiposity [18–21]. The cafeteria diet consists of highly energetic and highly palatable human foods along with chow diet to trigger diet-induced obesity in laboratory rodents [22]. In the absence of a purified high diet, the cafeteria diet is an alternative to induce obesity in that it prevents the use of very high intakes of a particular type of fat while inducing continuous hyperphagia and increased energy intake [23, 24] with a substantial amount of sugar and salt, which increases the appetite of rodents [25]. Buyukdere et al. [26] concluded that cafeteria diet is more suitable in inducing obesity and adiposity in young rats as compared to high fat diet.

*Piper nigrum* belongs to the Piperaceae family and is widely used in the food industry because of its culinary applications as well as health benefits, as spices and preservatives, and in the herbal medicine and cosmetic industry [27]. Both the berries and leaf of *P. nigrum* are served as hot spices and in some traditional clinics they are used in stimulating appetite in patients [28]. These *Piper* species are also used in folk medicine for the treatment of coughs, intestinal diseases, bronchitis, venereal diseases, colds, and rheumatism [28]. In some local communities in Africa, these spices are referred to as bush pepper in order to distinguish them from the common domesticated peppers [28]. In earlier studies, *Piper nigrum* demonstrated lipid-lowering effects and antioxidant capacity by prolonging the lag time for LDL + VLDL oxidation in the present of copper (II) ion [29–31]. Oral administration of aqueous extract of *P nigrum* leaf presented antioxidant defense and anti-atherogenic activity in hamsters fed atherogenic diet [31, 32]. In high-fat diet fed rats, the ethyl acetate and aqueous extracts of *Piper nigrum* seeds regulated body weight, percentage fat, and fat-free mass [33]. Phytochemical studies revealed the major bioactive molecules in *Piper nigrum* to include polyphenols, alkaloids, terpenoids, tannins, and oils while the major isolated compounds reported are piperine, piperidine, pellitorine, cepharadione, piperolactum, paprazine, and sylvamide [34]. Of these compounds, piperine has been widely studied to act on pharmacological systems [35]. Accumulated evidence from in vitro and in vivo studies has shown that piperine possesses anticancer [36, 37] and neuroendocrine modulator effects [38]. Some other studies have demonstrated the expectorant, antiflatulent, and cholesterol-lowering properties of *Piper nigrum* is due to its piperine [39, 40].

Most of the data generated on *Piper nigrum* are based on the berries and its chemical constituents with very little attention on the leaf that are often and commonly consumed as vegetables or spices. Secondly, there is no study that considered the effect of *Piper nigrum* leaf extract on cafeteria diet which mimics human feeding habit. Hence, the present study tests the hypothesis that hydro-ethanolic extract of the leaf of *Piper nigrum* (Piperaceae) possess antioxidant activity that prevents obesity and atherosclerosis development in cafeteria diet fed rats. The aim of this study was to evaluate the anti-obese, antioxidant, and cardioprotective effect of *Piper nigrum* (Piperaceae) leaf hydro-ethanolic extract on cafeteria diet fed rats. In order to verify the protective effect of *Piper nigrum* extract against obesity, and cardiovascular disease, we induce obesity by cafeteria diet as earlier described [41]. Hence, the experimental animals will be characterized by physiological changes which are similar to humans. Our findings would be informative on the health benefits of spices and/or medicinal food in oxidative stress and obesity related to the feeding habits.

## 2. Methods

### 2.1. Plant Collection and Preparation

Fresh leaves of *Piper nigrum* were collected in the locality of Djombé-Penja in the Littoral region, Cameroon in August 2017. No permission was needed for plant collection. The identification of the plant was done at the national herbarium of Yaoundé, Cameroon where the voucher specimen is kept under the number 2528 SRFK. 4 kg of fresh leaf of *Piper nigrum* was cleaned and dried at room temperature away from sunlight and then crushed to obtain a fine powder. The powder (1.615 kg) was macerated with 6.5 L of hydro-ethanolic solvent (70/30 ethanol and distilled water v/v) for 72 hours in a percolator. The mixture was filtered and the solvent evaporated with a rotavapor (Buchi R110) and then oven-dried at 40°C. The hydro-ethanolic extract (273.1 g) obtained gave an extraction yield of 6.82% which was then stored at −20°C and used for the study.

### 2.2. Phytochemical Screening

The extract was screened qualitatively for the identification of the different secondary metabolites in the hydro-ethanolic extract. The method described by Harbone [41] was used for the test of anthraquinones, saponins, sterols, terpenoids, steroids, flavonoids, coumarins, phenols, tannins, and glucosides. And for alkaloids, and anthocyanins, the Odebeyi and Sofowora method [42] was applied.

### 2.3. Composition of the Cafeteria Diet

Obesity was induced by a cafeteria diet composed according to the protocol of Darimont et al. [43]. The composition of the standard laboratory chow and the cafeteria (experimental) diet are presented in Table 1. These were the two types of diets used in this study: the normal and cafeteria diet.

### 2.4. Experimental Animals and Treatment

This study was carried out in line with the practice and principles of the institution on the use of experimental animals respecting the 2011 Guide for the Care and Use of Laboratory Animals, 8th edition, and the Animal Welfare Act. Thirty male Wistar Albino rats 6–7 weeks old and weighing between 123 g and 130 g obtained from the animal house of the Institute of Medical Research and Medicinal Plants Studies (IMPM), Yaoundé, Cameroon, were used for this study. The rats were housed in wire meshed (3 per cage) cages and maintained at 24 ± 2°C temperature and a cycle of 12 : 12 hours light/dark with free access to food and water at all time. After adaptation into the laboratory conditions for one week, the rats were randomly divided into 5 groups of 6 rats each (still maintained in 3 per cage) and fed for five weeks as follows:

- Group 1: Normal control (Nor.), administered standard diet
- Group 2: Negative control (Neg.), administered cafeteria diet
- Group 3: (Ator.): cafeteria diet + atorvastatin 10 mg/kg
- Group 4: (E250): cafeteria diet + Hydro-ethanolic extract 250 mg/kg
- Group 5: (E500): cafeteria diet + Hydro-ethanolic extract 500 mg/kg

All animals were fed with cafeteria diet except the animals of group 1 which was fed with standard diet and the feeding lasted five weeks. Alongside feeding, the animals were administered distilled water (Nor. and Neg.), atorvastatin (Ator.), and plant extracts (E250 and E500) in a volume of 100 ml/kg by oral intubation.

### 2.5. Sacrifice of Rats and Samples Collection

At the end of the feeding period (5 weeks), the experimental animals were fasted for 16 hours then taken into a separate room where they were weighed and sacrifice under pentobarbital sodium anesthetized (humane pharmaceutical grade, 78 mg/kg of body weight administered intraperitonially) and blood collected by cardiac puncture in to heparinize tubes. In brief, when the animal was asleep after anaesthesia as determined by no withdrawal of limb with pinching and by no response to a penlight shone in the eye, the animal was fixed on a dissecting board and incised mid-ventrally. Then, the chest part was opened by cutting the diaphragm and 5 ml of blood removed from the left ventricle (close to the surface of the chest) by cardiac puncture with a 5 ml syringe (21 *G* needle). In cases where the animal's heart was still beating after cardiac puncture, extra anaesthesia was administered to sacrifice the animal. The blood collected was centrifuged at 3000 rpm for 15 min at 4°C to separate the plasma which was stored in aliquots at −20°C for further biochemical analysis. The liver, kidney, heart, aorta, visceral fat, subcutaneous fat, and epididymal fat were carefully collected, rinsed with 0.9% NaCl, dried, and immediately weighed. Then a portion of liver, kidney, and heart was excised, and 10% w/v homogenate was prepared in ice-cold Tris-HCl 50 mM solution, respectively, and centrifuged at 3000 rpm for 25 min at 4°C. The supernatant obtained was used for the estimation of lipid peroxidation (malondialdehyde) and antioxidant activity (catalase, superoxide dismutase, and glutathione).

### 2.6. Determination of Body Weight, Lee Index, and Metabolic Efficiency Index

During the period of treatment, body weight of each animal was measured once per week in order to follow the development of over weight of the animals. The Lee index was calculated using the final body weight and nasal-anal length as in the equation below (equation (1)) [44]. Rats with Lee index (LI) higher or equal to 300 (Li ≥ 300) were considered obese.

(1) $$\begin{array}{c} \text{LI}=\left(\frac{\sqrt[{3}]{\text{body weight}}}{\text{nasal}-\text{anal length}\left(\text{cm}\right)}\right)\times 1000. \end{array}$$

The metabolic efficiency (ME) is the measure of how the body utilizes fat as an energy source. That is the body's ability to conserve its fat reserves or to mobilize them in the form of energy. This index was calculated as follows (equation (2)) [45]:

(2) $$\begin{array}{c} \text{ME}=\frac{\text{body weight gain}}{\text{food intake}}. \end{array}$$

### 2.7. Determination of Food and Water Consumption

The evaluation of the average of food and water intake per rat was recorded daily by subtracting the quantity of remaining food every day from the initial quantity provided the previous day.

### 2.8. Biochemical Parameters Analysis

Plasma concentration of transaminases, glucose, urea, creatinine, bilirubin, protein, triglycerides (TG), total cholesterol (TC), and HDL cholesterol (HDL-C) were all estimated through colorimetric methods with commercially available test kits according to the manufacturer's recommendations. The level of LDL-cholesterol (LDL-C) was calculated by subtracting the HDL cholesterol levels from the total cholesterol levels.

The atherogenic index (AI) (equation (3)) was calculated by using the method of Bais et al. [46].

(3) $$\begin{array}{c} \text{AI}=\frac{\left[\text{CT}\right]-\left[\text{HDL}\right]}{\left[\text{HDL}\right]}. \end{array}$$

### 2.9. Antioxidant Parameters Analysis

Antioxidant analysis was performed on the homogenate of different organs: liver, kidneys, and heart. The supernatant was used for the estimation of malondialdehyde (MDA) according to the protocol described by Wilbur et al. [47]. Glutathione (GSH) was assessed using Ellman's method [48], superoxide dismutase (SOD) activity was determined using the method described by Misra and Fridovich [49], and catalase activity was assessed using the method describe by Sinha [50].

### 2.10. Histological Examination

The aorta collected from all animals was rinsed and fixed in 10% buffered formalin, embedded in paraffin, and cut into 5 *μ*m sections that were stained with hematoxylin and eosin. These were observed under a microscope at 100x magnification. The histomorphometry of the aorta was evaluated with Image J software version 1.49.

### 2.11. Statistical Analysis

The data was analysed statistically using the GraphPad Prism 7.00 software. The results were expressed as mean ± SEM of 6 animals. The values were compared using the One-Way Analysis of Variances (ANOVA) test followed by the Tukey multiple comparison test. The differences were considered significant at *p* < 0.05.

## 3. Results

### 3.1. Phytochemical Screening of Hydro-Ethanolic Extract *Piper nigrum* Leaf

The results of the phytochemical screening of the hydro-ethanolic extract of the *Piper nigrum* leaf are summarized in Table 2. A plethora of secondary metabolites were identified, including gallic tannins, alkaloids, bound anthraquinones, phenolic compounds, flavonoids, and coumarins. The extract tested negative for terpenoids, anthocyanins, saponins, and glucosides.

### 3.2. Effects of Hydro-Ethanolic Extract of *Piper nigrum* Leaf on Food and Water Consumption

**Figure 1:**
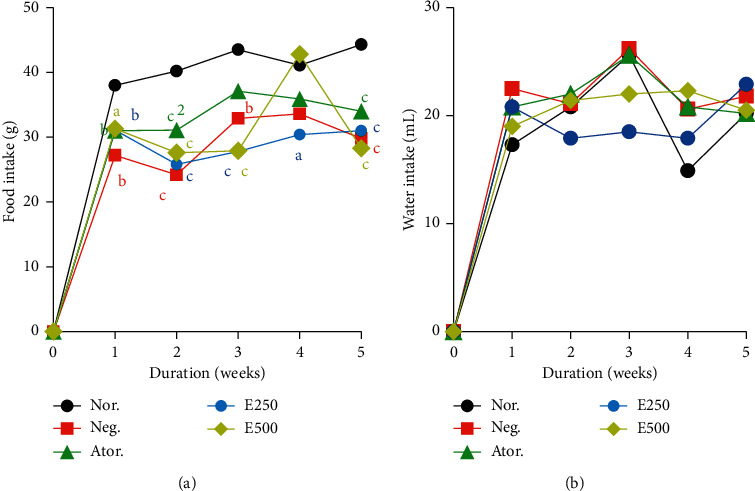
Effects of hydro-ethanolic extract of *Piper nigrum* leaf on food (a) and water (b) consumption.

Figure 1 presents the effects of the hydro-ethanolic extract of *Piper nigrum* leaf (E250 and E500 mg/kg) on food and water consumption of experimental animals. A significant decrease in food intake (*p* < 0.001) in the negative control group (untreated group) was observed compared to normal control group (Figure 1(a)). All groups of animals had an increase of water intake in the first week which slightly dropped in the second week. The pick of water consumption was attained at the third week for the normal and negative control animals and the atorvastatin (Ator.) administered group (Figure 1(b)). Amongst the plant extract treated groups, it was the E250 that had the lowest water consumption.

Each point represents the mean ± SEM, *n* = 6. *a*=*p* < 0.05, *b*=*p* < 0.01, *c*=*p* < 0.001 significant differences from the normal control; 1=*p* < 0.05, 2=*p* < 0.01, 3=*p* < 0.001 significant differences from the negative control; Nor.: normal control; Neg.: negative control; Ator.: atorvastatin (10 mg/kg); E250 and E500: animals fed with cafeteria diet and treated with hydro-ethanolic extract of *Piper nigrum* leaf at doses of 250 and 500 mg/kg.

### 3.3. Effects of Hydro-Ethanolic Extract of *Piper nigrum* Leaf on Lee Index, Metabolic Efficiency Index, and Atherogenic Index

With respect to the different indexes, no significant difference was found between the normal and the negative control groups (Table 3). However, a significant decrease in the atherogenic index (AI) of 57.06% (*p* < 0.01) was observed in the E500 treated group when compared to the negative control. No significant difference in Lee's index was observed across groups. Animals in the negative control group had significantly high metabolic efficiency (ME) of 73.02% (*p* < 0.001) compared to the normal control group. This increase was significantly inhibited to 30.44% (*p* < 0.01) and 44.70 (*p* < 0.001) in groups treated with atorvastatin and E500, respectively.

Each value represents mean ± SEM, *n* = 6. *a*=*p* < 0.05, *c*=*p* < 0.001 significant differences from the normal control; 2=*p* < 0.01; 3=*p* < 0.001 significant differences from the negative control. Nor.: normal control; Neg.: negative control; Ator.: atorvastatin (10 mg/kg); E250 and E500: animals fed with cafeteria diet and treated with hydro-ethanolic extract of *Piper nigrum* leaf at doses of 250 and 500 mg/kg. LI : Lee index, ME : metabolic efficiency, AI : atherogenic index.

### 3.4. Effects of Hydro-Ethanolic Extract of *Piper nigrum* Leaf on Body Weight Gain in Cafeteria Diet Fed Rats

**Figure 2:**
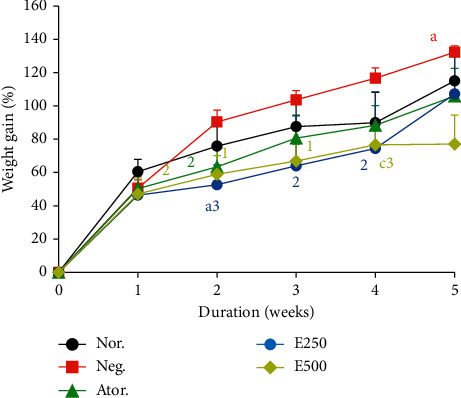
Effects of hydro-ethanolic extract of *Piper nigrum* leaf on body weight variation.

Figure 2 shows the effects of the hydro-ethanolic extract of *Piper nigrum* leaf on the weight change of rats fed with a cafeteria diet during an experimental period of five weeks. Feeding with cafeteria diet induced a significant increase in body weight in the negative control group of up to 30% (*p* < 0.05) compared to the normal control group in the fourth week of feeding. No significant difference was noted between groups at week one. In the second week of treatment, E250, E500, and atorvastatin significantly inhibited the increase in body weight of 41.81% (*p* < 0.001), 34.84% (*p* < 0.01), and 29.86% (*p* < 0) 01), respectively. At the third and fourth week of treatment, E250 significantly (*p* < 0.05) inhibited the effect of cafeteria diet in body weight by 38.46% and 36.41% and E500 inhibited by 35.67% (*p* < 0.05) and 34.52%, respectively. At the 5th week of treatment, while the body weight of the different groups continues to increase at different rates, the E500 group showed a significant inhibition of 42% (*p* < 0.001) compared to the negative control. Overall, the 250 mg/kg and 500 mg/kg extract resulted in a greater inhibition in weight gain in rats throughout the experiment.

Each point represents the mean ± SEM, *n* = 6. *a*=*p* < 0.05, *c*=*p* < 0.001; significant differences from the normal control; 1=*p* < 0.05, 2=*p* < 0.01, 3=*p* < 0.001 significant differences from the negative control; Nor.: normal control; Neg.: negative control; Ator.: atorvastatin (10 mg/kg); E.250 and E500: animals fed with cafeteria diet and treated with hydro-ethanolic extract of *Piper nigrum* leaf at doses of 250 and 500 mg/kg.

### 3.5. Effects of Hydro-Ethanolic Extract of *Piper nigrum* Leaf on the Relative Weight of Fats and Organs

**Figure 3:**
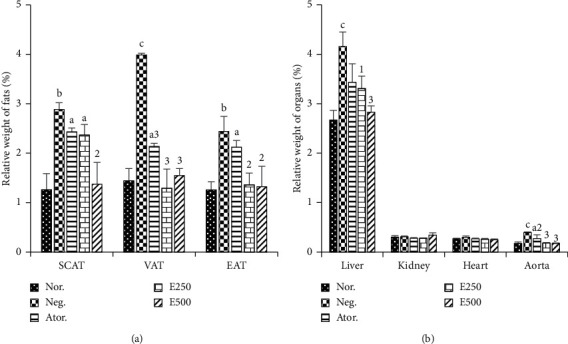
Effects of hydro-ethanolic extract of *Piper nigrum* leaf on adipose tissues and organs.

Figure 3(a) and 3(b) show the effects of the hydro-ethanolic extract of *Piper nigrum* leaf on the relative weight of fats and organs of experimental animals, respectively. A significant increase in the relative weight of subcutaneous (129.36%, *p* < 0.001), visceral (177.08%, *p* < 0.001), and epididymal adipose tissues (96.65%, *p* < 0.01) was observed in cafeteria diet fed rats (negative control). A significant decrease in the relative weight of visceral adipose tissue (VAT, 67.66%) and epididymal adipose tissue (EAT, 44.26%) was observed in the E250 group compared to the negative control. The E500 equally decreased the percentage of subcutaneous, VAT and EAT by 52.59% (*p* < 0.01), 61.15% (*p* < 0.001), and 45.90% (*p* < 0.01) compared to the negative control. Overall, the relative weight of adipose tissues of the animals treated with the doses of 250 and 500 mg/kg of the extract was comparable to those of the normal group.

Feeding cafeteria diet significantly increased the relative weight of the liver of 55.80% (*p* < 0.001) as indicated in the negative control group compared to the normal control group. *P nigrum* extract significantly decreased the relative weight of the liver of the order 20.43% (*p* < 0.05) and 39.18% (*p* < 0.001) in the groups treated with 250 mg/kg and 500 mg/kg, respectively. Similarly, a significant increase in the relative weight of the aorta in the negative control group (126.28%) was observed compared to the normal group. However, administered extracts significantly inhibited the increase in aorta weight to 54.29% (*p* < 0.001) and 55.80% (*p* < 0.001), respectively, in the group receiving the doses of 250 mg/kg and 500 mg/kg. No significant difference was found in the relative weights of kidney and heart of different groups of animals.

Each bar represents the mean ± SEM, *n* = 6. *a*=*p* < 0.05, *b*=*p* < 0.01, *c*=*p* < 0.001 significant differences from the normal control; 1=*p* < 0.05, 2=*p* < 0.01, 3=*p* < 0.001 significant differences from the negative control; Nor.: normal control; Neg.: negative control; Ator.: atorvastatin (10 mg/kg); E.250 and E500: animals fed with cafeteria diet and treated with hydro-ethanolic extract of *Piper nigrum* leaf at doses of 250 and 500 mg/kg. SCAT: subcutaneous adipose tissue; VAT: visceral adipose tissue; EAT: epididymal adipose tissue.

### 3.6. Effects of Hydro-Ethanolic Extract of *Piper nigrum* Leaf on Plasma Lipid Profile

Results of the effects of hydro-ethanolic extract of *Piper nigrum* leaf on lipid profile parameters in cafeteria diet fed rats are presented in Table 4. The cafeteria diet significantly increased the triglyceride, total cholesterol, LDL-C, and HDL-C concentrations in the order of 50.44% (*p* < 0.001); 66.90% (*p* < 0.001), 111.39%, and 41.86% (*p* < 0.05), respectively. Treatments with Ator, E250, and E500 significantly decreased the triglyceride levels of 39.41% (*p* < 0.001), 35.29% (*p* < 0.001), and 47.52% (*p* < 0.001), respectively. Only the E250 and E500 significantly decreased the total cholesterol level by 15.12% (*p* < 0.05) and 33.61% (*p* < 0.001), respectively, compared to the negative control. A significant decrease in LDL-C of 63.01% (*p* < 0.01) was noted in the E500 group compared to the negative control.

Each value represents mean ± SEM, *n* = 6. *a*=*p* < 0.05, *b*=*p* < 0.01, *c*=*p* < 0.001 significant differences from the normal control; 1=*p* < 0.05, 2=*p* < 0.01, 3=*p* < 0.001 significant differences from the negative control. Nor.: normal control; Neg.: negative control; Ator.: atorvastatin (10 mg/kg); E.250 and E500: animals fed with cafeteria diet and treated with hydro-ethanolic extract of *Piper nigrum* leaf at doses of 250 and 500 mg/kg.

### 3.7. Effects of Hydro-Ethanolic Extract of *Piper nigrum* Leaf on Plasma Blood Glucose Levels and of Hepatic and Renal Function

Table 5 presents the effects of hydro-ethanolic extract of *Piper nigrum* leaf on plasma blood glucose levels and some markers of hepatic (ALAT and ASAT) and renal (bilirubin, creatinine, and urea) function in cafeteria diet fed rats. Cafeteria diet significantly increased the transaminase activity (ALAT and ASAT), glucose, total protein, and bilirubin contents, respectively, of 64.51% (*p* < 0.001), 84.81% (*p* < 0.001), 83.76% (*p* < 0.001), 97.35% (*p* < 0.001), and 20.68% (*p* < 0.05) with respect to the normal control.

Each value represents the mean SEM, *n* = 6. *a*=*p* < 0.05, *b*=*p* < 0.01, *c*=*p* < 0.001 significant differences from the normal control; 1=*p* < 0.05, 2=*p* < 0.01, 3=*p* < 0.001 significant differences from the negative control. Nor.: normal control; Neg.: negative control; Ator.: atorvastatin (10 mg/kg); E.250 and E500: animals fed with cafeteria diet and treated with hydro-ethanolic extract of *Piper nigrum* leaf at doses of 250 and 500 mg/kg.

Also, an increase in creatinine and urea concentration of 88.8% and 2.64% was observed in negative control group compared to the normal group. However, E250 significantly decreased ALAT and ASAT activity by 38.23% (*p* < 0.001) and 50.68% (*p* < 0.05), respectively, while E500 significantly decreased by 57.10% (*p* < 0.001) and 49.31% (*p* < 0.001). The E250 animals induced a significant inhibition in blood glucose levels of 16.74% (*p* < 0.001) compared to the negative control. The significant decreases in total protein levels of 57.86% (*p* < 0.001), 50.67% (*p* < 0.001), and 66.89% (*p* < 0.001) were observed in the E250 and E500 groups, respectively. Only the E500 animals showed a significant decrease in the level of bilirubin. Cafeteria diet significantly (*p* < 0.001) increased the concentration of creatinine as presented by the negative control. The effect of cafeteria diet on creatinine was significantly (*p* < 0.001) inhibited by the administration of *Piper* extract with E500 having the best effect. The effect of E500 was comparable to atorvastatin and the normal control.

### 3.8. Effects of Hydro-Ethanolic Extract of *Piper nigrum* Leaf on Lipid Peroxidation and Antioxidant Enzymes in Organs

**Figure 4:**
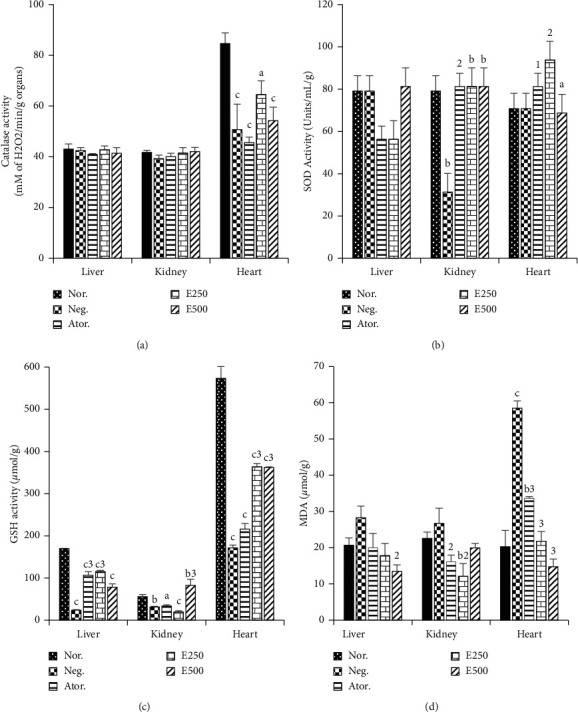
Effects of hydro-ethanolic extract of *Piper nigrum* leaf on some parameters of oxidative stress.

Figure 4(a) reveals a significant decrease in catalase activity in the heart tissue of cafeteria diet fed rats (40.14%, *p* < 0.001) compared to the normal control group. When *Piper* extracts were administered, they effectively prevented the collapse in catalase activity in the heart tissue, thus boosting the antioxidant activity. However, no significant difference between groups was observed in the liver and kidney catalase activity.

Cafeteria diet induced a significant decrease in SOD activity of 60.67% (*p* < 0.01) in the kidney of the negative control group but did not have any effect on the liver and heart SOD (Figure 4(b)). The increase in SOD activity in the kidney was inhibited significantly by atorvastatin (159.74%, *p* < 0.01) and the E250 and E500. In the heart homogenate, a significant increase in SOD activity of 85.61% (*p* < 0.05) and 114.15% (*p* < 0.01) was observed in atorvastatin and E250 groups compared to the negative control. No significant differences were found in the liver of the negative control and the other treated groups.

With respect to GSH, a significant decrease of 85.94% (*p* < 0.001), 44% (*p* < 0.01), and 70.15% (*p* < 0.001) was noted, respectively, in the liver, kidney, and the heart of the negative control group compared to the normal control group (Figure 4(c)). Compared to the negative control group, a significant increase in GSH levels of 347.69% (*p* < 0.001) and 381.17% (*p* < 0.001) was noted, respectively, in the liver of atorvastatin and E250. Only the E500 group showed a significant increase in the level of GSH (163.67%, *p* < 0.001) in the kidney compared to the negative control. In the heart, the E250 and E500 groups showed an increase in GSH level of 112.28% (*p* < 0.001) and 111.69%. (*p* < 0.001).

Measurement of lipid peroxidation in animal organs was quantified by the level of MDA in homogenates. Figure 4(d) indicates a significant increase in MDA level in heart homogenates of 188.17% (*p* < 0.001) in the negative control group compared to the normal control group. On the other hand, no significant difference was found in the homogenates of liver and kidneys of these two groups. Treatment with E500 induced a decrease in the level of MDA in the liver of 52.29% (*p* < 0.01) and in the kidney of 39.70% (*p* < 0.01) compared to the negative control group. In heart homogenates, a significant decrease in the MDA level of 62.73% (*p* < 0.001) and 74.87% (*p* < 0.001) was noted, respectively, in the groups treated with atorvastatin and with E250 and E500. The E250 extract had a better effect on restoring catalase and SOD activities than E500 in the heart tissues.

Each bar represents the mean ± SEM, *n* = 6. *a*=*p* < 0.05, *b*=*p* < 0.01, *c*=*p* < 0.001 significant differences from the normal control; 1=*p* < 0.05, 2=*p* < 0.01, 3=*p* < 0.001 significant differences from the negative control. Nor.: normal control; Neg.: negative control; Ator.: atorvastatin (10 mg/kg); E.250 and E500: animals fed with cafeteria diet and treated with hydro-ethanolic extract of *Piper nigrum* leaf at doses of 250 and 500 mg/kg.

### 3.9. Effects of the Hydro-Ethanolic Extract of *Piper nigrum* Leaf on the Architecture of the Aorta

**Figure 5:**
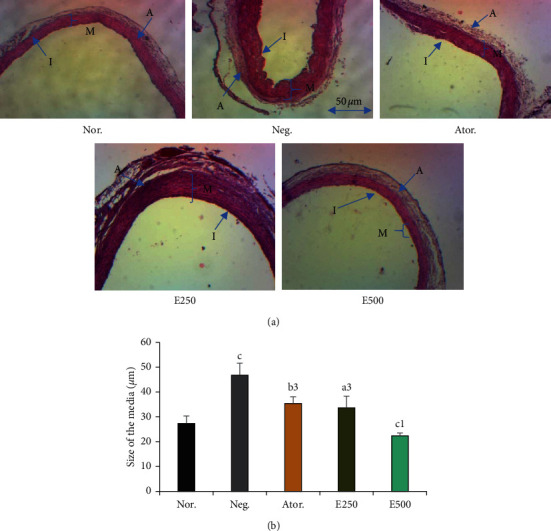
Effects of hydro-ethanolic extract of *P. nigrum* leaf on the architecture of aorta. (a) Hematoxylin-eosin 40X. (b) Histomorphometry data of the aorta under Image J software version 1.49.

Figure 5 shows the effects of the hydro-ethanolic extract of *Piper nigrum* leaf on the architecture of the aorta. The consumption of a cafeteria diet induced a significant thickening of the media and the structural disorganization of the intima in negative control group compared to normal control group (Figure 5(a)). The E250 and E500 groups showed a significant decrease in the thickness of the media. All groups exhibited a structural rearrangement of the architecture of the aorta which was comparable to the normal control group. The histomorphometry data of the aorta under Image J software presented in histograms (Figure 5(b)) are the extrapolated thickness of the aorta. The aorta of the negative control rats had the highest accumulation of fats measuring up to 46.78 ± 4.84 *μ*m compared to normal (27.37 ± 2.98 *μ*m). Atorvastatin reduced the thickening of the aorta to 35.38 ± 2.67 *μ*m while the *Piper* extracts reduced the thickening to 33.66 ± 4.70 *μ*m (E250) and 22.36 ± 1.14 *μ*m (E500). The effect of the plant extract was dose-related and comparable to that of atorvastatin.

Each bar represents the mean ± SEM, *n* = 6. *a*=*p* < 0.05, *b*=*p* < 0.01, *c*=*p* < 0.001 significant differences from the normal control; 1=*p* < 0.05, 3=*p* < 0.001 significant differences from the negative control. Nor.: normal control; Neg.: negative control; Ator.: atorvastatin (10 mg/kg); E.250 and E500: animals fed with cafeteria diet and treated with hydro-ethanolic extract of *Piper nigrum* leaf at doses of 250 and 500 mg/kg.

## 4. Discussion

The present study reported cafeteria diet-induced weight gain leading to obesity in negative control group compared to normal control group but with a reduction in food intake similar to earlier report by Bais et al. [46]. This justifies that weight gain does not necessarily depend on the amount of food consumed but more on the caloric intake as provided in the cafeteria diet whose consumption led to an increase in energy reserves and an increased weight gain [25, 43]. With cafeteria diet life style being embraced in modern times where people do not have time for a regular and/or organic food, this study provides a possible outcome in its consumption, and the importance of spices such as *P. nigrum* if added to a meal.

The overall increased relative weight of adipose tissue and organs, and the concentrations of total cholesterol, LDL-C, HDL-C, and triglyceride in cafeteria diet fed rats corroborated the results of Cabot et al. [51] and Meryem et al. [52]. This may be as a result of an excessive accumulation of fats in the tissues, linked to a very high bioavailability of lipids and the need for the body to store them in one way or another, without taking into account the lipotoxic effects generated by this excessive storage [51]. The dyslipidemia observed was directly related to the accumulation of fat in adipose tissue including visceral adipose tissue. According to Grundy et al. [53], the accumulation of fat promotes the acceleration of the flow of free fatty acids from the portal vein to the liver, which contributes to the hypersecretion of VLDL particles (very-low-density lipoproteins) accounting for the high plasma triglycerides concentrations observed in this study. The elevation of plasma triglyceride levels is generally accompanied by numerous disturbances in lipid metabolism. This is the case of the increased expression of the hydroxy-methyl-glutaryl Coenzyme-A (HMG-CoA) enzyme, increased LDL-cholesterol particle size, decreased HDL-C, increased atherogenic risk, and inhibited lipoprotein lipase activity [52, 54, 55]. The increase of HDL-C in negative control in the present study could be explained by the presence of phytoestrogens contained in soy (included in the diet) that promote the synthesis of HDL-C in the liver [56]. This effect of phytoestrogens varies from subject to subject and would be greater in individuals with excess cholesterol in the body [57].

Rats treated the with extract of *Piper nigrum* and atorvastatin presented an overall protective effect in weight gain and amelioration of lipid profile in cafeteria diet intoxication. The similarity in dietary intake between the negative control group and the groups treated with different doses of plant extract suggests that the extract had no anorectic effects that could justify the low rate of weight gain observed. The inhibition of weight gain may be attributed to the thermogenic effects of the plant and its action on the metabolism of fats. Indeed, *Piper nigrum* contains a plethora of secondary metabolites involved in many biochemical processes. This is the case of piperine, a potent alkaloid that inhibits lipid accumulation by stimulating metabolic enzymes such as lipoprotein lipase and adenosine monophosphate activated protein kinase (AMPK) that promote the use of lipids by peripheral tissues [30, 58]. It also contains phenolic compounds and flavonoids which would be involved in thermogenesis and decrease the expression of genes involved in weight gain and differentiation of pre-adipocytes into adipocytes [59]. In an earlier study, Ibrahim et al. [60] reported a higher total flavonoid, total phenolic, gallic acid, and rutin content in the leaves of *P. nigrum* compared to the seeds which may be responsible for the biological effect of the plant. Hence, consumption of the *Piper nigrum* leaf may be more beneficial to health than the seeds.

With regard to the different indexes, an increase in the atherogenic index and the metabolic efficiency was noted in the negative control group compared to the normal control group related to the development of obesity in these animals. According to Lossa et al. [61] and Bais et al. [46], this increase is linked to the consumption of the cafeteria diet similar to most high-calorie diets linked to an increase in metabolic efficiency and atherogenic risk. The animals treated with the different doses of the extract and atorvastatin showed a decrease in these different indexes compared to the negative control. The decrease in metabolic efficiency and the atherogenic index could be justified by an increase in energy expenditure [45, 62] and by the cardioprotective effects of the plant [63].

The blood glucose concentration and markers of renal and hepatic functions presented an increase in transaminases, glucose, urea, creatinine, bilirubin, and total proteins in cafeteria fed animals (negative control) compared to normal control. Earlier researchers have equally reported the effect of cafeteria diet on hyperglycaemia associated with insulin resistance or glucose intolerance [64], liver damage indicative of increased transaminases and bilirubin levels [46]. Equally, renal damage that results in increased levels of urea and creatinine and inflammatory phenomena has been reported in cafeteria diet intoxicated experimental animals [65, 66]. *Piper nigrum* extract and atorvastatin were able to prevent modification of all these parameters and maintained them towards normal. Hence, properties which are indicative of the hepatoprotective and nephroprotective activity of the plant related to bioactive compounds that possess antioxidant properties [67, 68]. Mir et al. [59] also linked the decrease in glucose concentration to flavonoids which possess strong antioxidant activities. However, the decrease in glucose concentration was not dose dependent as the E250 presented a better protective effect than E500. The high blood glucose concentration in E500 compared to E250 might have resulted from the effect of a by-product that interfered with its antihyperglycemic activity. The effect of *P nigrum* on glucose metabolism may be an important tool in the management of hyperglycaemia resulting from overweight and obesity.

Superoxide dismutase protects the tissues against reactive oxygen species by scavenging superoxide radical and producing hydrogen peroxide which is the substrate of catalase. Catalase then reduces hydrogen peroxide to oxygen and water. Reduced glutathione acts as a substrate of glutathione peroxidase and Glutathione-S-Transferases and yields water molecule and other organic hydroperoxides. Obesity have been reported to induce a collapse of the antioxidant defense mechanism characterized by diminishing activities of superoxide dismutase (SOD), catalase (CAT), and glutathione peroxidase (GPx) resulting in an accumulation of lipid peroxides and reactive oxygen species. [13]. Cafeteria diet consumption has been associated with obesity and oxidative stress in experimental animal by alteration in the catalase, SOD, glutathione, and MDA levels [30, 67]. Also, obesity has been reported to induce systemic oxidative stress, resulting in a collapse in the antioxidant defense system characterized by decreased activity of catalase, superoxide dismutase, reduced glutathione levels, and an increase in the MDA marker of lipid peroxidation [69]. In the present study, though the cafeteria diet significantly altered the antioxidant defense mechanism, it did not have a significant effect on liver and kidney CAT activity. However, the plant extract improved the antioxidant defense system by an increase in the antioxidant enzymes activity and a decrease in MDA accumulation at E250 and E500 dose levels. However, the E250 had a better effect of catalase and SOD activities in the heart tissue. The non-significant changes in CAT activity in liver and kidney tissues of obese rat may be attributed to increase in oxygen consumption by the adipose tissues and the change of CAT enzyme activity is dependent on oxygen consumption. Meanwhile, the heart tissue uses a large amount of fatty acids and glucose as energetic substrate and the final oxidation of these fuels occurs in the mitochondria by aerobic mechanisms, which justify the heart tissue susceptibility to oxidative stress. In a similar way, sixteen weeks fed high fat diet had no significant effect on liver and heart CAT activity in experimental rats [70]. The unexpected high SOD activity in the liver and the heart tissue may present false positive results considering the effect of cafeteria diet on the atorvastatin and the extract treated groups.

Histopathological analysis of the architecture of the aortic revealed a thickening of the media and structural disorganization of the intima in negative control animals following consumption of a cafeteria diet. Indeed, the hypercaloric diet induces dyslipidemia which later caused an accumulation of lipids mainly by the action of LDL-C in the arteries. These factors in turn lead to structural alteration of the vessel and ultimately to the formation of atheromatous plaques [71]. The extract at different doses prevented the thickening of the media and induced a structural rearrangement of the intima. It could be justified by the antioxidant properties of the plant that would limit the oxidation of LDL-cholesterol and the progression of atherosclerosis closely related to cardiovascular complications [32, 72].

## 5. Conclusion

This study was designed to evaluate the preventive effect of hydro-ethanol extract of *Piper nigrum* leaf on cafeteria diet-induced obesity in rats. The cafeteria diet consumed by animals during the experimental period led to an increase in body weight and relative weight gain of the organs, dyslipidemia with an increased risk of atherogenesis, liver and kidney damage, and oxidative stress. Hence, such dietary lifestyle may be detrimental to human health. Co-administration with *Piper nigrum* leaf extract inhibited the effect of the cafeteria diet which justifies the health benefits of *Piper nigrum*. This may be important in the human physiology when overwhelmed with oxidative stress.

## Figures and Tables

**Table 1:** Composition of different diets (g/g of ingredient).

Ingredients	Standard diet (g)	Energetic value (kcal)	Cafeteria diet (g)	Energetic value (kcal)
Corn flour	530	1852.35	278	971.61
Wheat flour	128	463.36	55	199.10
Cheese	—	—	110	249.70
Biscuit	—	—	110	498.30
Soy	310	130.51	160	67.36
Pork liver pate	—	—	110	262.72
Banana chips	—	—	50	265.40
Peanuts	—		50	294
Chocolate	—		50	275
Vitamin complex (multi vitamin plus)	20	—	20	—
Bone powder	10	—	5	—
Salt	2	—	2	—
Total		2446.22		3083.19

**Table 2:** Phytochemical screening of *Piper nigrum* hydro-ethanolic extract.

Compounds	Results
Gallic tannins	+
Alkaloids	+
Anthraquinones (bounds)	+
Phenolic compounds	+
Flavonoids	+
Coumarins	+
Terpenoids	-
Anthocyanins	-
Saponins	-
Glucosides	-

(+): present, (-): absent.

**Table 3:** Effects of *Piper nigrum* on Lee index, metabolic efficiency index, and atherogenic index.

	Nor.	Neg.	Ator.	E250	E500
LI	0.32 ± 0.01	0.31 ± 0.007	0.31 ± 0.009	0.31 ± 0.008	0.30 ± 0.004
ME	0.786 ± 0.02	1.36 ± 0.04c	0.946 ± 0.012	1.09 ± 0.05	0.752 ± 0.033
AI	1.76 ± 0.12	1.90 ± 0.18	1.45 ± 0.26	1.56 ± 0.22	0.82 ± 0.01a2

**Table 4:** Effects of hydro-ethanolic extract of *Piper nigrum* leaf on lipid profile.

	Nor.	Neg.	Ator.	E250	E500
TG (mg/dL)	113 ± 0.54	170 ± 6,02^c^	103 ± 0.76^3^	110 ± 2.37^3^	89.2 ± 2.9^c3^
Cholesterol (mg/dL)	71.3 ± 4.79	106 ± 6.67^c^	91.5 ± 4.46^b^	101 ± 4.41^b^	79 ± 3.42^2^
HDL-C (mg/dL)	25.8 ± 1.09	36.6 ± 1.73^a^	40.5 ± 3.42^c^	40 ± 2.39^b^	43.2 ± 2.07^c^
LDL-C (mg/dL)	22.9 ± 4.07	35.4 ± 6.44	30.4 ± 6.89	39 ± 3.44	17.9 ± 1.9

**Table 5:** Effects of hydro-ethanolic extract of *Piper nigrum* leaf on markers of hepatic and renal function.

	Nor.	Neg.	Ator.	E250	E500
ALAT (U/I)	12.4 ± 0.49	20.4 ± 1.54^c^	12.6 ± 0.70^3^	12.5 ± 0.69^3^	8.75 ± 1.47^3^
ASAT (U/I)	23.7 ± 1.7	43.8 ± 3.09^c^	30.3 ± 1.57^1^	21.6 ± 2.63^3^	22.2 ± 2.94^3^
Glucose (mg/dl)	117 ± 1.18	215 ± 4.27^c^	164 ± 2.49^c3^	179 ± 6.30^c3^	203 ± 5.71^c^
Urea (mg/dl)	38.9 ± 0.60	39.8 ± 0.76	37.3 ± 0.40	36.8 ± 0.76	37.8 ± 0.76
Total protein (g/dl)	8.31 ± 0.20	16.4 ± 1.26^c^	7.01 ± 0.75^3^	6.91 ± 0.39^3^	8.09 ± 0.50^3^
Creatinine (mg/dl)	2.25 ± 0.47	4.25 ± 0.75^c^	2.5 ± 0.28^3^	3.0 ± 0.40^b1^	2.67 ± 0.66^3^
Bilirubin	14.5 ± 0.27	17.5 ± 1.46^a^	17.5 ± 0.45^a^	16.0 ± 0.46	13.6 ± 0.41^2^

## Data Availability

The datasets used and analysed during the study are included in the manuscript without any restriction.

## Abbreviations

AI: Atherogenic index
ALAT: Alanine aminotransferase
AMPK: Adenosine monophosphate activated protein kinase
ANOVA: Analysis of variance
ASAT: Aspartate aminotransferase
Ator.: Atorvastatin
EAT: Epididymal adipose tissue
Eq: Equation
g: Gram
GSH: Glutathione
HCl: Hydrochloric acid
HDL: High-density lipoprotein
HMG-CoA: 3-hydroxy-3-methylglutaryl-Co-enzyme A
IMPM: Institute of Medical Research and Medicinal Plants Studies
kg: Kilogram
LDL: Low-density lipoprotein
LI: Lee index
MDA: Malondialdehyde
ME: Metabolic efficiency index
mg/dL: Milligrams per decilitre
mg/kg: Milligrams per kilogram
NaCl: Sodium chloride
Neg.: Negative control
Nor.: Normal control
*P. nigrum*: *Piper nigrum*
RNS: Reactive nitrogen species
ROS: Reactive oxygen species
SCAT: Subcutaneous adipose tissue
SEM: Standard error of mean
SOD: Superoxide dismutase
TC: Total cholesterol
TG: Triglyceride
VAT: Visceral adipose tissue
VLDL: Very-low-density lipoprotein
WHO: World Health Organization.
